# The Turkish Medicines and Medical Devices Agency: Comparison of Its Registration Process with Australia, Canada, Saudi Arabia, and Singapore

**DOI:** 10.3389/fphar.2018.00009

**Published:** 2018-01-25

**Authors:** Emel Mashaki Ceyhan, Hakki Gürsöz, Ali Alkan, Hacer Coşkun, Oğuzhan Koyuncu, Stuart Walker

**Affiliations:** ^1^Centre for Innovation in Regulatory Science, London, United Kingdom; ^2^School of Pharmacy and Pharmaceutical Sciences, Cardiff University, Cardiff, United Kingdom; ^3^Turkish Medicines and Medical Devices Agency, Ankara, Turkey

**Keywords:** Turkish regulatory review, TITCK, SFDA, TGA, Health Canada, HSA

## Abstract

**Introduction:** Regulatory agency comparisons can be of more value and facilitate improvements if conducted among countries with common challenges and similar health agency characteristics. A study was conducted to compare the registration review model used by the Turkish Medicines and Medical Devices Agency (Türkiye Ilaç ve Tibbi Cihaz Kurumu; TITCK) with those of four similar-sized regulatory agencies to identify areas of strength and those requiring further improvement within the TITCK in relation to the review process as well as to assess the level of adherence to good review practices (GRevP) in order to facilitate the TITCK progress toward agency goals.

**Methods:** A questionnaire was completed and validated by the TITCK to collect data related to agency organizational structure, regulatory review process and decision-making practices. Similar questionnaires were completed and validated by Australia's Therapeutic Goods Administration (TGA), Health Canada, Singapore's Health Science Authority (HSA), and the Saudi Arabia Food and Drug Administration (SFDA).

**Results:** The TITCK performs a full review for all new active substance (NAS) applications. Submission of a Certificate of Pharmaceutical product (CPP) with an application is not required; however, evidence of approval in another country is required for final authorization by the TITCK. Pricing data are not required by the TITCK at the time of submission; however, pricing must be completed to enable products to be commercially available. Mean approval times at the TITCK exceeded the agency's overall target time suggesting room for improved performance, consistency, and process predictability. Measures of GRevP are in place, but the implementation by the TITCK is not currently formalized.

**Discussion:** Comparisons made through this study enabled recommendations to the TITCK that include streamlining the good manufacturing practice (GMP) process by sharing GMP inspection outcomes and certificates issued by other authorities, thus avoiding the delays by the current process; removing the requirement for prior approval or CPP; introducing shared or joint reviews with other similar regulatory authorities; formally implementing and monitoring GRevP; defining target timing for each review milestone; redefining the pricing process; and improving transparency by developing publicly available summaries for the basis of approval.

## Introduction

With the support of the World Health Organization (WHO) and the involvement of regulatory authorities, the entire pharmaceutical regulatory and authorization approval systems were reshaped after the 1960s to define the minimum standards for drug development and marketing authorizations as well as to promote harmonization of pharmaceutical regulations across countries to ensure the timely access of patients to comparatively safe and effective medicines.

While each country has its own national requirements, it is well recognized that individual health authorities have different expertise, competencies and knowledge that could be of value to other countries by comparing the various review models and sharing best practices. However, a comparison of an agency in a country with an emerging pharmaceutical market with mature health agencies such as the US Food and Drug Administration (FDA) and European Medicines Agency (EMA) may lead to an unreasonable assessment due to the different characteristics and competencies these agencies possess. For example, the FDA has the largest number of reviewers compared with other health agencies and a broad scope that includes many areas other than pharmaceutical products (United States Food and Drug Administration, 2016). The EMA is a central networking organization agency with 28 member countries representing a population of almostfive hundred million people, and the review and decision-making processes within the EMA involve many experts from across Europe and the use of a model that depends on a rapporteur and co-rapporteur (European Medicines Agency, 2016). Regulatory agency comparisons can be of more value and facilitate improvements if conducted among countries with common challenges and similar health agency characteristics. Agencies from jurisdictions with emerging pharmaceutical markets may have an interest in comparing themselves with other similarly sized mature regulatory authorities such as Health Canada and Australia (Hashan et al., 2016).

With a population of 80 million, Turkey is the second largest pharmaceutical market in Central/Eastern Europe (United States Department of Commerce, 2016). Between 2002 and 2011, healthcare expenditure per capita grew by 150% (Fortune Türkiye, 2016) and in 2015, Turkey spent 142.8 billion Turkish lira ($US39.4 billion) or 5.5% of its gross domestic product on healthcare. Of this amount, 19.4% or 27.5 billion Turkish lira ($US7.6 billion) was spent on pharmaceuticals (United States Department of Commerce, 2016). Turkey's 67 manufacturing centers and 300 corporations were responsible for exports to 160 countries in 2016, largely to the European Union (EU), Middle East and North Africa (MENA) and Commonwealth of Independent States (CIS) countries. In 2015, the Higher Planning Council issued the “Turkish Pharmaceutical Industry Strategic Action Plan of 2015–2018,”aiming to greatly increase pharmaceutical research and development, production and management, including a target for the domestic production of 60% of pharmaceuticals and 20% of medical devices consumed in Turkey by 2018 (Fortune Türkiye, 2016).

Universal healthcare was introduced in Turkey in 2004 and the Turkish government is responsible for the majority (77%) of healthcare spending; however, the role of private insurance is expected to continue to increase in Turkey as is healthcare spending in general, spurred by a growing, aging population and the increasing prevalence of long-term health conditions such as diabetes and heart disease (United States Department of Commerce, 2016). To ease the pressure of government spending, Turkey instituted a reference drug pricing system and a fixed exchange rate in 2009, resulting in the pricing of drugs to be one of the lowest among similar countries. This pricing system increased the affordability and access to the healthcare system, but has also decreased pharmaceutical industry profitability and resulted in some product shortages (Fortune Türkiye, 2016). In 2015, legislation ended the use of the fixed rate, allowing reference prices to be converted at 70% of the previous year's average Euro/Lira exchange rate (Schonherr, 2016). Furthermore, the updated pricing communique introduced flexible pricing pathways for certain products such as life-saving, critical and orphan drugs to be decided by the Pricing Evaluation Committee (Fiyat Degerlendirme Komitesi; FDK) within the Türkiye Ilaç ve Tibbi Cihaz Kurumu (TITCK).

Affiliated with the Ministry of Health in 2012, TITCK, the Turkish Medicines and Medical Devices Agency is the governmental regulatory authority responsible for regulation, evaluation, inspection, control and monitoring of human medicinal products, medical devices and cosmetics in Turkey. In Turkey, the registration review process of pharmaceutical products is conducted in accordance with the “*Registration Regulation of Human Medicinal Products*,” which sets forth the principles, procedures, and policies regarding the registration of medicines (Ministry of Health, 2005). The main goals and focus areas of the Turkish health authority in the past decades have included alignment with international standards and the development of a robust high-quality regulatory health agency comparable to those of other mature developed health agencies, in order to ensure the timely access of patients to medicines.

To date, comparative data to demonstrate the performance of the TITCK registration review model with other developed and emerging countries of similar sizes and characteristics have not yet been identified. Therefore, there was a need for such a study, as the TITCK wishes to become a reference agency in the region. Similar studies have been carried out for Saudi Arabia (Hashan et al., 2016) the Jordan FDA (Haqaish et al., 2016) compared with Australia, Canada and Singapore, as well as Australia and Denmark (Aagaard et al., 2008). Therefore, a study was conducted to compare the registration review model in Turkey with Australia, Canada, Saudi Arabia and Singapore. The study aimed to identify areas of strength and those requiring further improvement within the TITCK in relation to the review process as well as to assess the level of adherence to good review practices (GRevP) in order to facilitate the TITCK progress toward agency goals.

## Materials and methods

### Study participants

The regulatory health authorities responsible for the regulation and review process for pharmaceutical products in five countries were included in this study namely; Australia's Therapeutic Goods Administration (TGA), Health Canada, Singapore's Health Science Authority (HSA), and the Saudi Arabia Food and Drug Administration (SFDA) as well as the Turkish Medicines and Medical Devices Agency (TITCK). The countries were selected by the TITCK on the basis of the comparability of the size of the agencies, the time that they had been established, the fact that they carry out a full review as well as the patient population they serve.

### Data collection process

The questionnaire designed and used in this study was completed by the TITCK to collect data related to the regulatory review process for new active substances (NASs), including the marketing authorization applications submission dates, registration dates, and the overall review and approval timelines. The questionnaire was designed by the Centre for Innovation in Regulatory Science (CIRS), London, UK and previously used to evaluate the regulatory process for new medicines in jurisdictions with emerging pharmaceutical markets, to identify the regulatory aspirations, barriers, problems, and priorities related to the review of new medicines that can have an impact on their availability to patients (McAuslane et al., 2009). The questionnaire was designed to enable mapping of the process flow and the internal parameters that influence the progress of regulatory review and to understand the decision-making processes, implementation of GRevP, and review outcomes in countries like Saudi Arabia (Hashan et al., 2016).

This standard mapping facilitated the collection of important information and allowed the data to be illustrated in a common format to simplify comparisons among regulatory agencies. The questionnaire was divided into three parts, in which the first part aimed to provide details of the TITCK organizational structure and resources and to explore the review model(s) used for the scientific assessment of medicines. The second part of the questionnaire aimed to explore the review and approval process for NASs within the agency through a standard process map, which allowed for the description of review processes and included common definitions. The third part of the questionnaire documented the activities that contribute to the quality of the decision-making process and measures adopted by the TITCK to build quality into the assessment and registration process in order to improve consistency, transparency, and timeliness. Following the completion of the questionnaire by the TITCK, data were transferred into a Microsoft Word report, enabling auditing, discussion, and any necessary modifications by the TITCK.

Similar questionnaires had also been completed and validated within the same time frame by Australia's Therapeutic Goods Administration (TGA), Health Canada, Singapore's Health Science Authority (HSA), and the Saudi Arabia Food and Drug Administration (SFDA) (Hashan et al., 2016). These data were also transferred into Microsoft Word reports and sent to the regulatory authorities for auditing, correction, and further comments. The consistent format and standardized terminology of the questionnaire enabled the compilation of important information about the structure, processes, and practices of international regulatory agencies for the purpose of comparison.

## Results

### Comparative assessment of regulatory review processes and milestones

**Figure 1:**
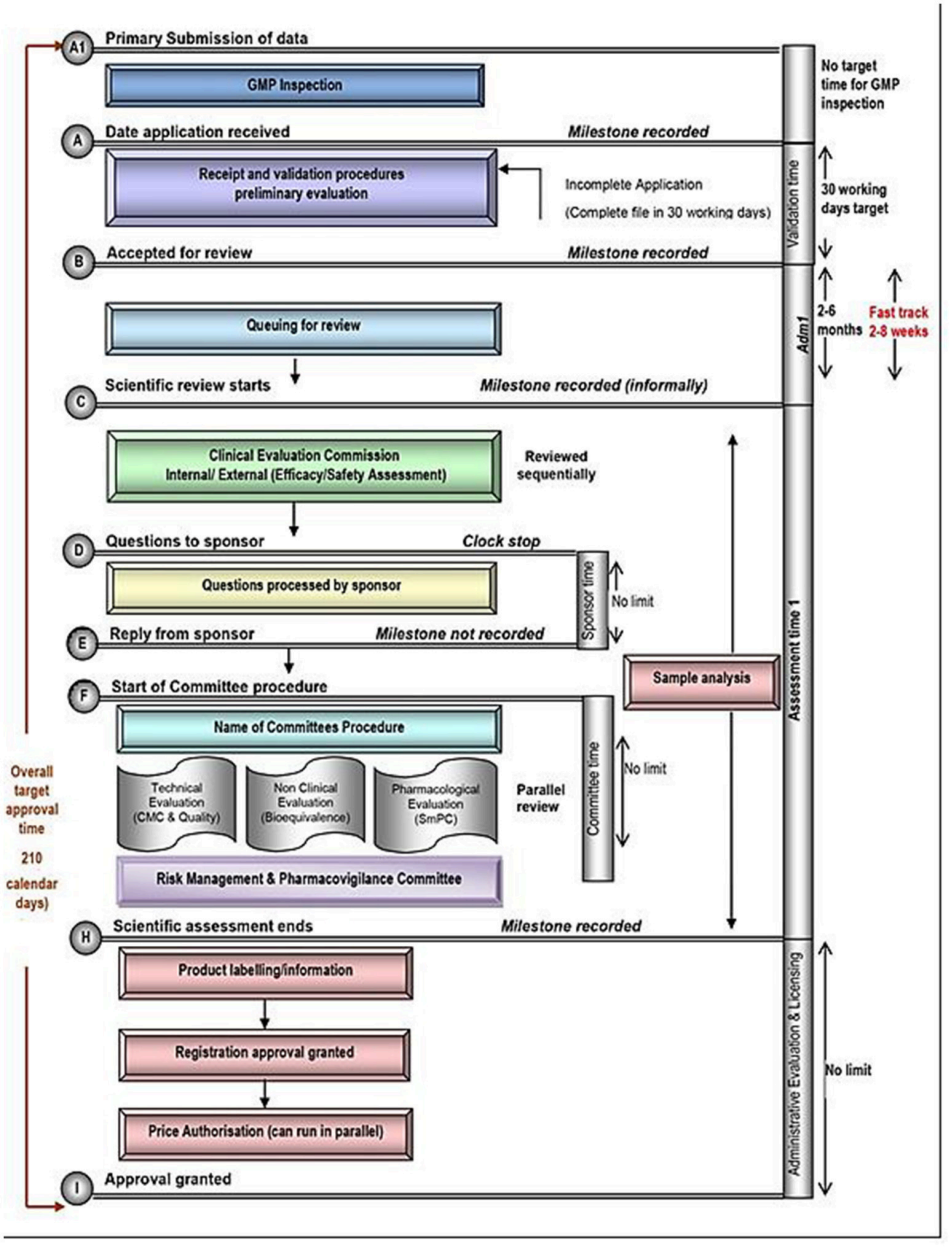
Registration process map for Turkey.

**Figure 2:**
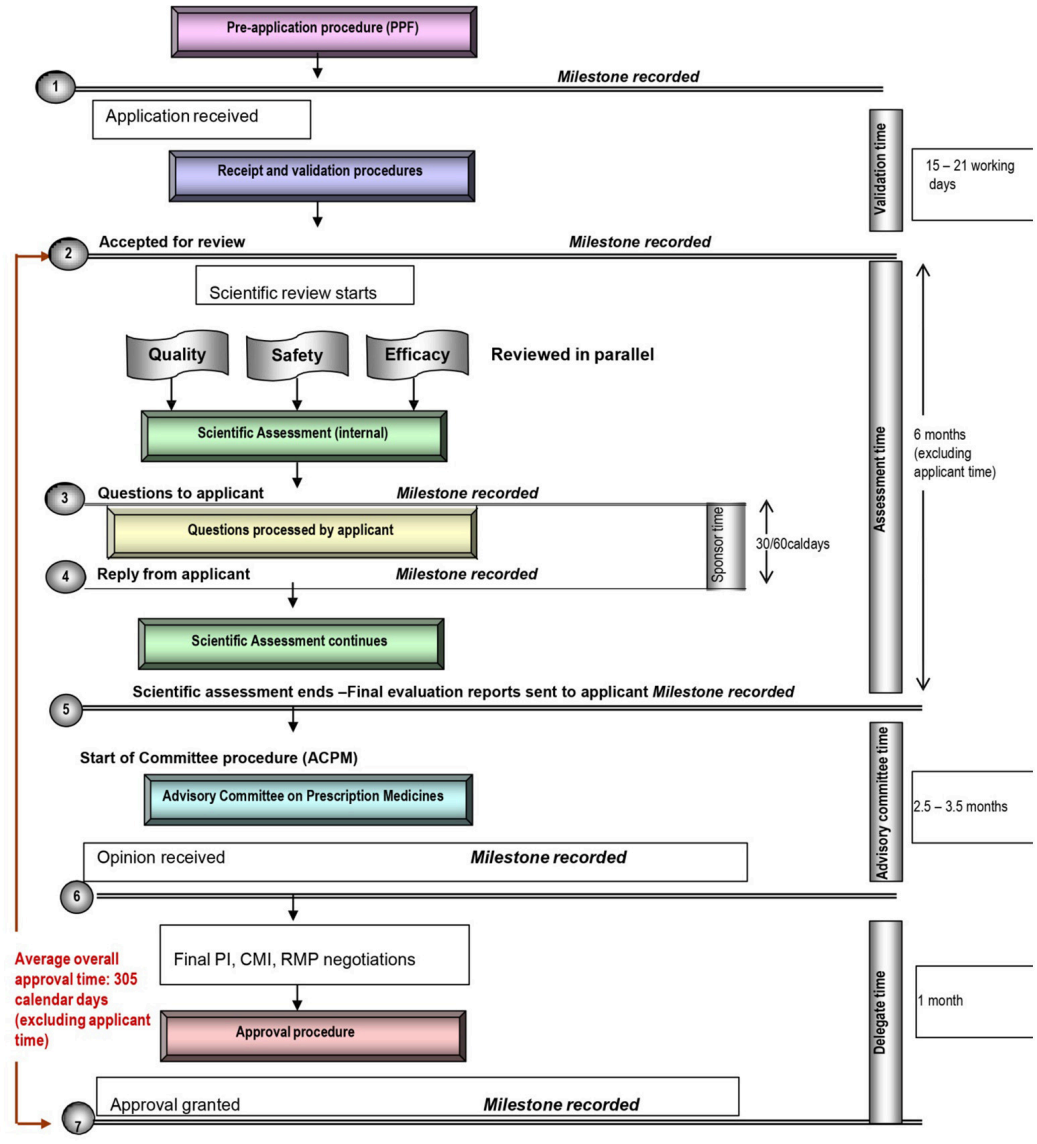
Registration process map for Australia.

**Figure 3:**
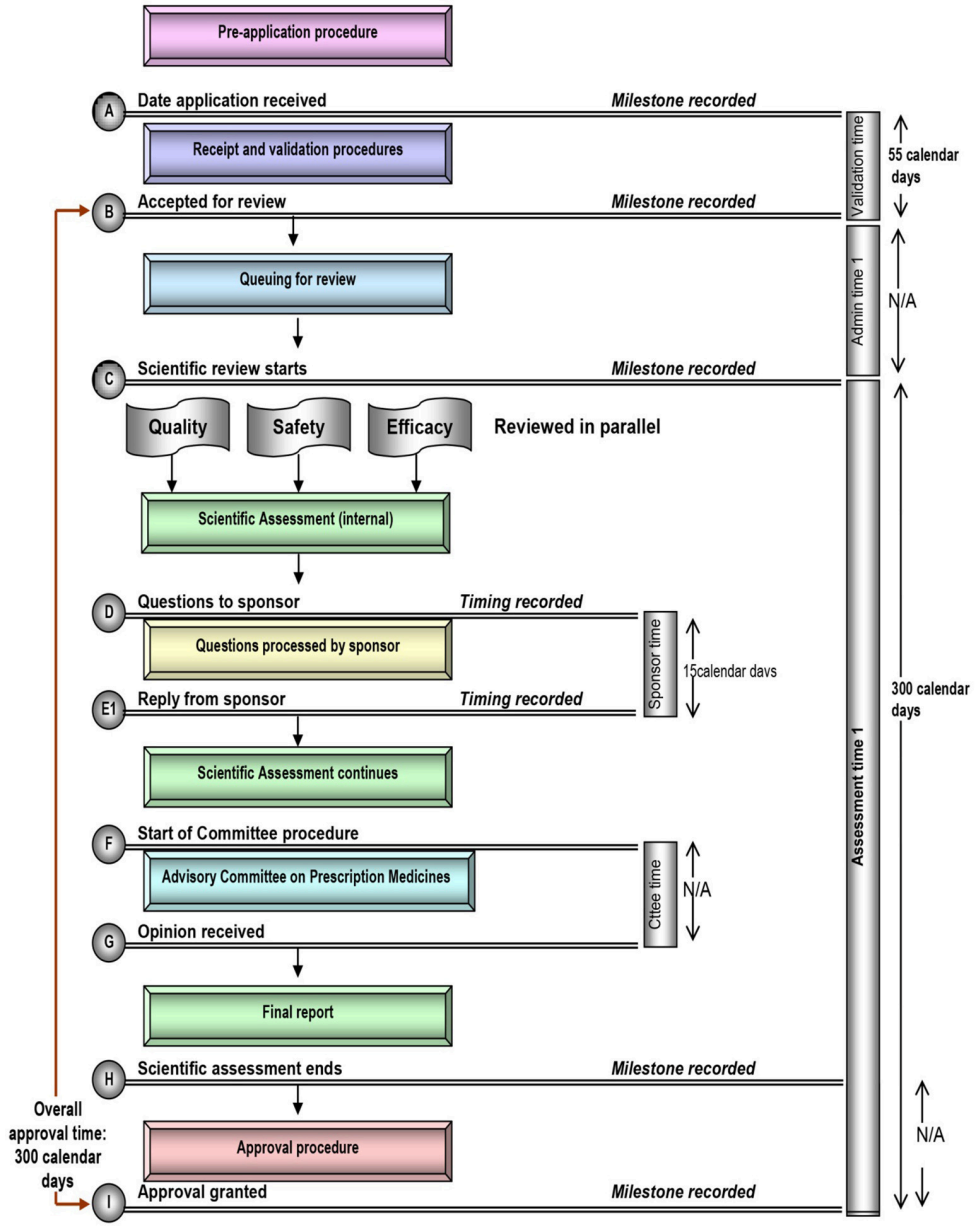
Registration process map for Canada.

**Figure 4:**
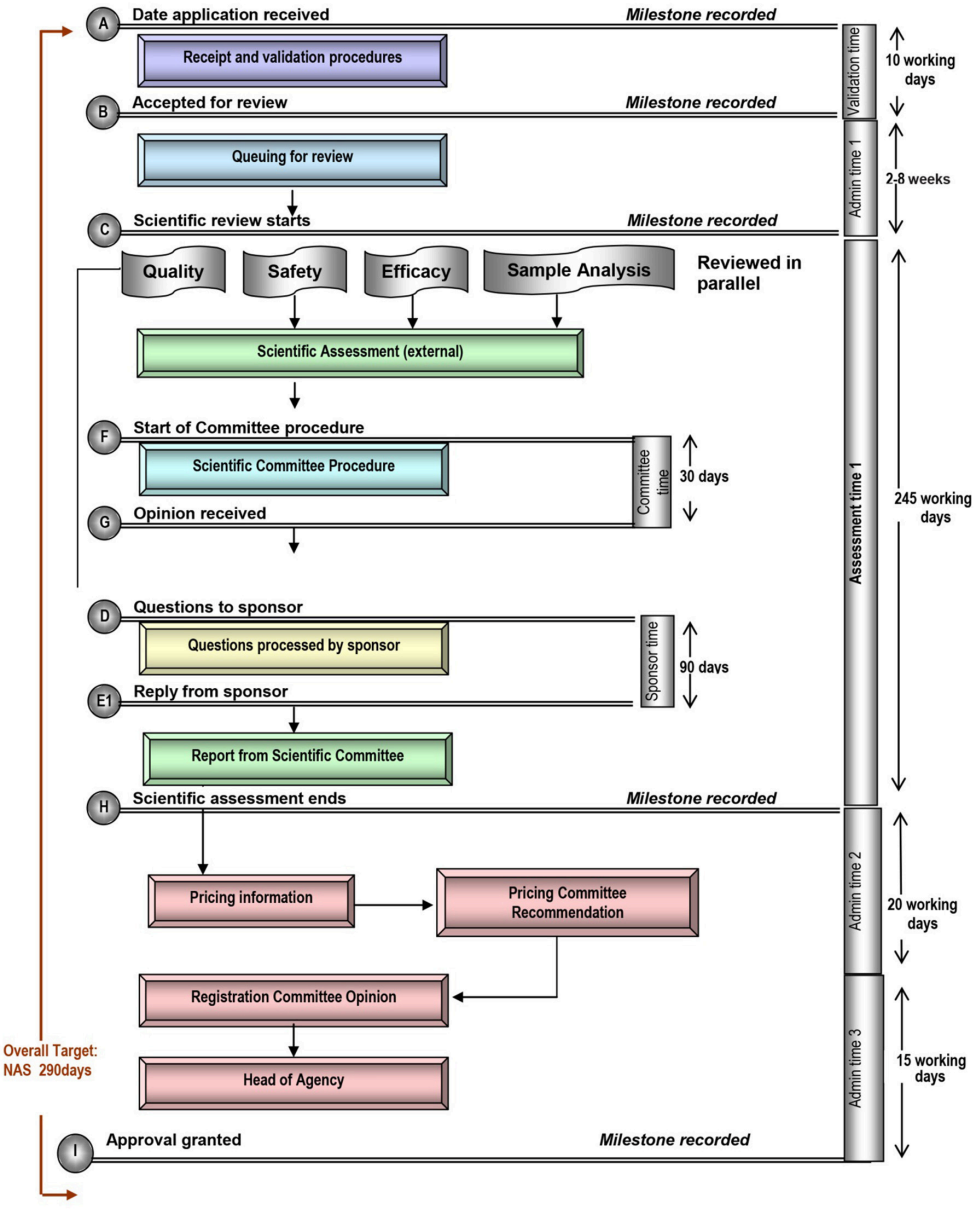
Registration process map for Saudi Arabia.

**Figure 5:**
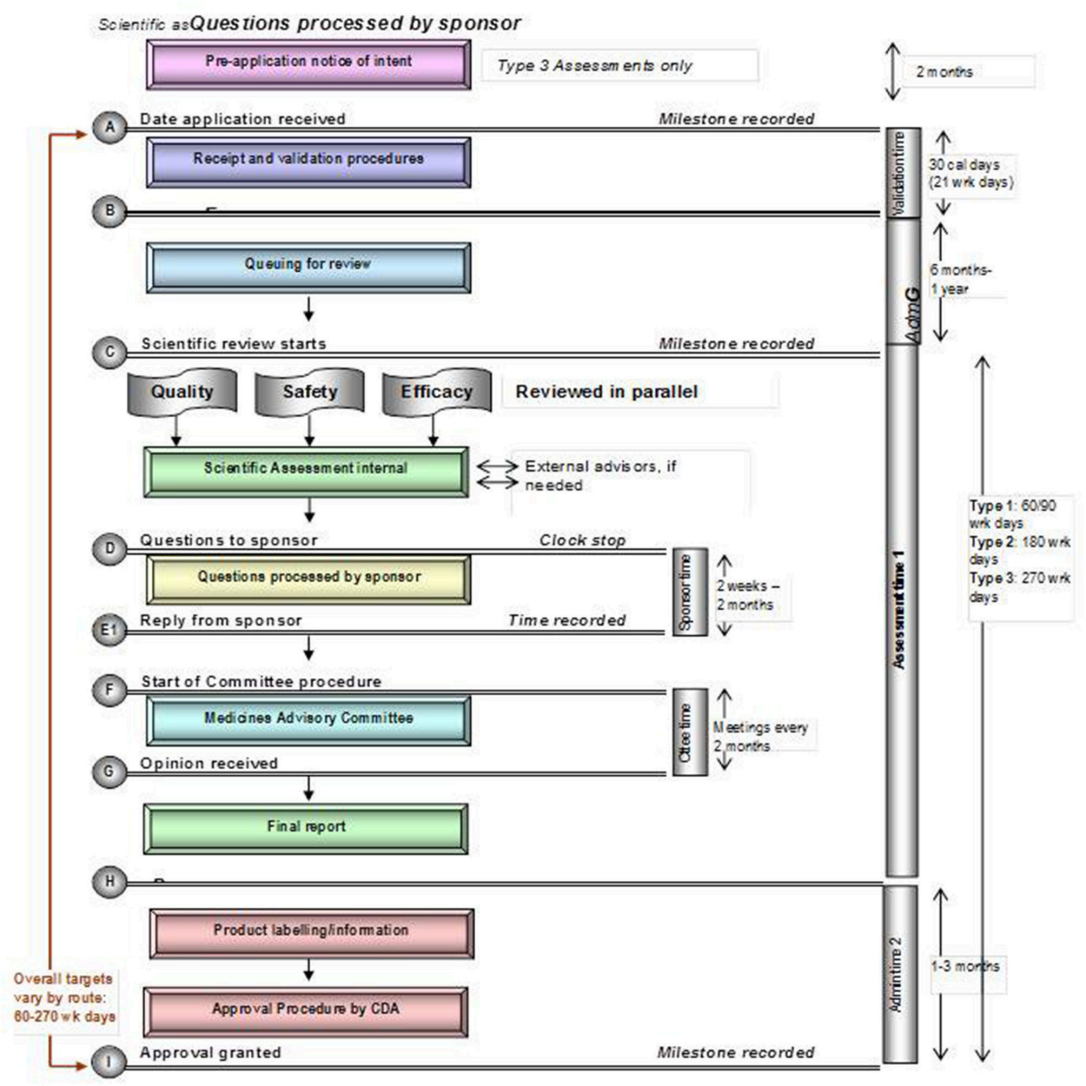
Registration process map for Singapore.

Each of these five mid-sized regulatory health authority have similar goals for regulating the pharmaceutical industry and establishing the marketing authorization standards and requirements to ensure the timely access of patients to medicines while safeguarding their safety, quality, and efficacy. Nevertheless, regulatory authorities demonstrate a number of differences within their review systems in terms of processes, timelines and review practices. Process maps of the five countries are presented in a standardized format, which enables appropriate comparisons (Figures 1–5).

#### Review model

Many regulatory health authorities apply a different level of data assessment, corresponding to the type of product being reviewed and/or its worldwide regulatory status. According to McAuslane and colleagues, there are three basic types of scientific regulatory review of products (McAuslane et al., 2009).

The *type 1 verification model* is generally used to reduce duplication of review effort since it requires that the product be authorized by two or more recognized reference agencies. The regulatory agency is only responsible to verify and validate the application for local marketing to ensure that it conforms to that agreed in the reference authorization(s).

*The type 2, the abridged assessment model* conserves resources by not re-assessing scientific supporting data that has been reviewed and approved by at least one reference or competent regulatory agency and includes an abridged independent review of the product in terms of its use under local conditions. In *the types 3A and 3B full assessment models* the agency carries out a complete scientific review and evaluation of the supporting scientific data (quality, pre-clinical, and clinical) for a major application. While pre-registration by a reference agency is required for type 3A assessment, it is not required for type 3B.

The TITCK performs a full review for all new active substance applications and a marketing authorization application for a new active substance can be submitted in Turkey prior to any approval in the world. However, because evidence of approval in other countries like the EU or US must still be submitted prior to the final approval by the TITCK, the agency review type is considered to be type 3A.

The SFDA, TGA, HSA, and Health Canada also all utilize a full assessment model (Table 1). The SFDA, which requires that a certificate of pharmaceutical product (CPP) be submitted with the application for final marketing authorization is type 3A, whilst the TGA, Health Canada, and HSA do not require a CPP for submission and are type 3B. It should be noted, however, that the TGA can conduct a type 2 abridged review to conserve resources if requested by the sponsor and if the product has been approved by two or more reference agencies and HSA conducts an abridged review if the product has been approved by one or more reference agencies, or a type 1 verification model if the product has been approved by two or more reference agencies.

**Table 1:** Models of assessment of the five agencies and extent of the scientific review.

**Type of review model**	**Turkey**	**Australia**	**Canada**	**Saudi Arabia**	**Singapore**
Verification review (type I)	✘	✘	✘	✘^a^	✓^b^
Abridged review (type II)	✘	✓^c^	✘	✘	✓^d^
Full review (type III)	✓	✓	✓	✓	✓
**EXTENT OF SCIENTIFIC REVIEW**					
**1. Chemistry, manufacturing, and control (CMC) data**					
Extensive assessment	✓	✓	✓	✓	✓
**2. Nonclinical data**					
Extensive assessment	✓	✓	✓	✓	✓^e^
**3. Clinical data**					
Extensive assessment	✓	✓	✓	✓	✓
**ADDITIONAL INFORMATION OBTAINED (WHERE APPROPRIATE)**					
Other agencies' internal review reports	✓	✓	✓	✘	✘
Reports on the internet	✓	✓	✓	✓	✓
General internet search	✓	✓	✓	✓	✓

a *The SFDA recently announced that it will conduct a verification review if the product has been approved by the EMA and the FDA*.

b *Only if the product has been approved by two or more reference agencies*.

c *Only if requested by the sponsor and if the product has been approved by two or more reference agencies*.

d *Only if the product has been approved by one or more reference agencies*.

e *Only for biological and biosimilar products*.

#### Data requirements

The TITCK requires full clinical and efficacy data for the application, which must be submitted in the common technical document (CTD) format with Modules 1–5 for scientific data. The TITCK performs a complete assessment of these data and additionally performs a structured benefit-risk assessment and examines the influence of ethnic factors and the differences in medical culture and practice, national disease patterns, and unmet medical needs even though sufficient data on these criteria are not always supplied in all applications.

**Figure 6:**
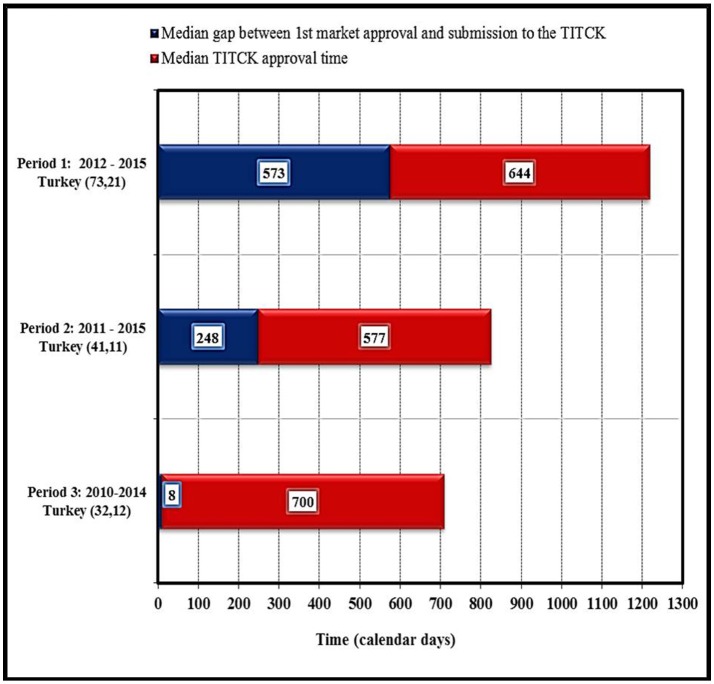
Impact of current good manufacturing processes on approval timelines of NASs by TITCK (industry data).

Most of the quality elements of the application are assessed by the TITCK through the good manufacturing practices (GMP) accreditation process, in which a complete GMP application is submitted for evaluation and all involved sites are physically inspected. The GMP accreditation process is a pre-requisite for all new marketing authorization and type 2B site-related chemistry, manufacturing, and control (CMC) applications. As a result, the start of the review process can be delayed by 12 to 18 months. An exception to this process is made only for life-saving and critical products categorized as highly prioritized products (category one), for which the GMP accreditation process can be conducted in parallel to the review process to save time and accelerate patients' access to these products (Türkiye Ilaç ve Tibbi Cihaz Kurumu, 2016). The impact of the current GMP process was evaluated in this study by looking at NAS approval dates for three different periods based on data provided by pharmaceutical companies: first period from 2012 to 2015, the second from 2011 to 2015 and third period from 2010 to 2014. The figures in brackets (Figure 6) indicate the number of NASs as well as the number of companies. These data identified that the duration of the gap between first market approval of NASs and TITCK submission for the second period (2011–2015) was 248 calendar days (8 months) compared with only 8 calendar days for NAS approved in the third period from 2010 to 2014. This indicated an increasing delay and gap in the NAS applications to the TITCK after first approval anywhere in the world since 2010. The delay could be attributed to the introduction of the Turkish GMP regulation in 2010 where many companies were either hesitant or not able to submit their NAS applications due to the GMP requirements, which was later on amended in 2012 (Figure 6). Thus, in the first and later period (2012–2015) the median delay has increased to 573 calendar days. Subsequently, the median TITCK approval time varied during these three periods monitored from 700 calendar days in period 3 to 577 calendar days in period 2 to 644 calendar days in period 1.

In comparison, the GMP process in Saudi Arabia and Australia may be completed by the submission of a copy of a GMP certificate issued from a reference agency (Australian Government Department of Health Therapeutic Goods Administration, 2016), whilst in Singapore, the HSA may conduct an inspection in parallel to the review process according to the ICH GMP and/or Pharmaceutical Inspection Co-operation Scheme (PIC/S) guidelines (Singapore Health Sciences Authority, 2016).

All the comparative authorities in this study require full datasets for the pharmaceutical CMC, non-clinical, and clinical sections of a dossier and conduct a detailed assessment of all three sections for full review. However, the assessments conducted by the HSA depend on the type of review it conducts, allowing the conservation of resources for use in the review of medicines associated with a high risk for the Singapore population. At the TITCK, the sequence of the scientific review of the application can vary according to the status of the application, and several committee reviews can run in parallel or sequentially depending on the product's submission status with the EMA or FDA. Thus, in the case of a parallel marketing authorization application submission with EU or FDA, the registration review process starts with the technical assessment and then proceeds with the clinical evaluation in order to consider the EMA or FDA clinical decision or opinion. In comparison, the review of quality, safety, and efficacy data are conducted in parallel at the other four regulatory agencies in this study.

External experts are used on an ad hoc basis by the TGA, HSA, and SFDA, whereas Health Canada does not use external experts for dossier review. The TITCK use a list of120 external experts who, in addition to the internal experts, attend weekly different committee meetings held within the TITCK and are mainly responsible for providing a detailed assessment report and recommendations as well as a clinical opinion on the product.

Pricing data are not required by the TITCK at the time of submission; however, pricing negotiation starts during the registration review and licensing process. The pricing process can be conducted in parallel and independently to the registration review after the main clinical assessment is completed. Nevertheless, registration approval can be obtained prior to pricing approval since pricing approval is only required to be completed prior to the sales and importation permission applications, which enable products to be commercially available. Of the five agencies, only the SFDA requires information related to pricing as part of the marketing authorization dossier.

#### Target and approval times

According to TITCK regulations, the overall approval target timeline is 210 calendar days, 180 calendar days for a prioritized accelerated review and 150 calendar days for highly prioritized products. However, *prioritized accelerated review* and *highly prioritized* are not defined in the regulation. Nevertheless, the draft updated registration regulation provides more clarity on those definitions. In any case, the actual approval timelines are much longer in practice than those stated in the TITCK regulation. The TITCK review process consists of the following common steps: validation of the submitted dossier, scientific assessment, company response and final authorization. The TITCK target time for the validation is 30 working days. The mean times for NAS marketing authorizations applications approved 2013, 2014, and 2015 were 350, 340, and 270 working days, respectively.

In comparison, the overall target approval time for the SFDA, which conducts a type 3A review is 420 calendar days or 290 working days. For TGA, Health Canada, and HSA, which all conduct type 3B reviews, overall target approval times are 305, 355, and 395 calendar days respectively, although it should be noted that HSA conducts mainly abridged reviews (Table 1). During this same time period review times for SFDA were shorter than the target time, review times for TGA for 2012–2015 exceeded specified targets, and review times for Health Canada were approximately on target.

### Comparative assessment of good review practices

This study identified the different quality metrics that have been implemented by the five agencies with the aim of comparing the practices in place to ensure quality, transparency, and predictability of the regulatory review process. All of the studied agencies have GRevP in place but implement them informally, except for Health Canada, which has a programme for formal use.

#### Quality measures

The quality measures evaluated in this comparative study included the availability and use of an internal quality policy, GRevP, standard operating procedures (SOPs) for assessors, assessment templates, a quality assurance department, the use of scientific committees, and the use of shared and joint reviews with other agencies. The TITCK have six of these seven measures in place, namely, an internal quality policy, GRevP system, SOPs for assessors, assessment templates, a dedicated quality assurance department and a scientific committee (Table 2). In comparison, SFDA and TGA also each have six of the seven measures in place, whereas Health Canada and HSA employ five. Additionally, Health Canada, TGA, SFDA, and HSA occasionally conduct shared or joint reviews with other regulatory authorities.

**Table 2:** The quality measures implemented by the five agencies.

**Measure**	**Regulatory authority**				
	**Turkey (6/7)**	**Australia (6/7)**	**Canada (5/7)**	**Saudi Arabia (6/7)**	**Singapore (5/7)**
Internal quality policy	✓	✓	✘	✘	✘
Good review practice system	✓ *(Informally)*	✓ *(Informally)*	✓ *(Formally)*	✓ *(Informally)*	✓ *(Informally)*
Standard operating procedures for guidance of assessors	✓	✓	✓	✓	✓
Assessment templates	✓	✓	✓	✓	✓
Dedicated quality department	✓	✘	✘	✓	✘
Scientific committee	✓	✓	✓	✓	✓
Shared and joint reviews	✘	✓ *(Occasionally)*	✓ *(Occasionally)*	✓^a^	✓ *(Occasionally)*

a *Shared and joint review with the Gulf Cooperation Council countries*.

#### Transparency and communication

Information communicated by regulatory health authorities to the public and relevant stakeholders could include: feedback on submitted dossiers, technical staff contact information, pre-submission scientific advice, official guidelines, the ability to track the progress of applications summary of the grounds of approval, approval times, advisory committee meeting dates and the approval of products. The TITCK provides four of these nine types of communication, including information provided to the general public regarding approved products and product labeling, feedback to industry on submitted dossiers (at the validation step only), official regulatory guidelines to assist industry and industry can track applications based on ad hoc contacts with the TITCK. The TITCK also has an internal electronic system to track applications; however, the system cannot be accessed by applicants nor does it provide information regarding review timelines. Official pre-submission advisory meetings are also not provided by the TITCK, but such meetings can be conducted by request on an informal, ad hoc basis, depending on the case under review. In comparison, the SFDA employs five of the communication parameters, HSA six, Health Canada eight, and TGA all nine (Table 3).

**Table 3:** Transparency and communication parameters in the five agencies.

**Measure**	**Regulatory authority**				
	**Turkey (4/9)**	**Australia (9/9)**	**Canada (8/9)**	**Saudi Arabia (5/9)**	**Singapore (6/9)**
Feedback to industry on submitted dossiers	✓	✓	✓	✘	✘
Details of technical staff to contact	✘ *(But some details available)*	✓	✓	✘	✓
Pre-submission scientific advice to industry	✘	✓	✓	✓	✓
Official guidelines to assist industry	✓	✓	✓	✓	✓
Industry can track progress of applications	✓ *(Based on ad hoc contact)*	✓	✓	✓	✓
Summary of grounds on which approval was granted	✘	✓	✓	✘	✘
Approval times	✘	✓	✓	✓	✓
Advisory committee meeting dates	✘	✓	✘	✘	✘
Approval of products	✓	✓	✓	✓	✓

Of the five agencies, TITCK, SFDA, and HSA do not publish a summary basis of approval, while SFDA and HSA do not give feedback to the industry on the submitted dossier. Neither the TITCK nor the SFDA share information that would be needed to contact their technical staff during the review, possibly because of concern that sponsors might attempt to influence reviewers.

#### Continuous improvement initiatives

The continuous improvement initiatives assessed in this study included external and internal quality audits, tracking systems and the review of assessors' and stakeholders' feedback. These study results indicated that the TITCK does have an internal tracking system to track the different milestones of applications through the various review stages. The TITCK conducts internal quality audits through its dedicated internal quality department and therefore has four of the five continuous improvement processes, while also Australia and Singapore have four, Health Canada has three and Saudi Arabia engages in all five continuous improvement processes (Table 4).

**Table 4:** Continuous improvement initiatives in the five agencies.

**Measure**	**Regulatory authority**				
	**Turkey (4/5)**	**Australia (4/5)**	**Canada (3/5)**	**Saudi Arabia (5/5)**	**Singapore (4/5)**
External quality audits	✘	✘	✘	✓	✘
Internal quality audits	✓	✓	✓	✓	✓
Internal tracking systems	✓	✓	✓	✓	✓
Reviews of assessors' feedback	✓	✓	✘	✓	✓
Reviews of stakeholders' feedback	✓	✓	✓	✓	✓

#### Training and education

The type of training and continuing education that can enhance the review process includes international workshops, external and in-house courses, on-the-job training, lectures by external speakers, induction training, sponsorship of postgraduate degrees as well as placements and secondments. The TITCK apply all of the training and education elements except for the provision of induction training for new employees and assessors, which the agency includes as part of on-the-job training. In comparison, the SFDA incorporates seven training and education elements, lacking only the availability of in-house courses, whereas Australia, Canada and Singapore employ all eight (Table 5).

**Table 5:** Training and education in the five agencies.

**Measure**	**Regulatory authority**				
	**Turkey (7/8)**	**Australia (8/8)**	**Canada (8/8)**	**Saudi Arabia (7/8)**	**Singapore (8/8)**
International workshops/conferences	✓	✓	✓	✓	✓
External courses	✓	✓	✓	✓	✓
In-house courses	✓	✓	✓	✘	✓
On-the-job training	✓	✓	✓	✓	✓
External speakers invited to the authority	✓	✓	✓	✓	✓
Induction training	✘	✓	✓	✓	✓
Sponsorship of post-graduate degrees	✓	✓	✓	✓	✓
Placements and secondments in other regulatory authorities	✓	✓	✓	✓	✓

#### Enablers and barriers to good-quality decision making

This study identified TITCK perceptions regarding its positive qualities and the major impediments it faces in carrying out the review of new medicines. The agency indicated that the availability of a pool of high-caliber employees and scientific committee experts as well as the opportunity to build close relationships with other international regulatory authorities, to share good decision-making processes and practices are factors that make a major contribution to the effectiveness and efficiency of the TITCK review. Whilst other agencies provided a diverse set of enablers as part of their questionnaire responses, there was some consistency among all five countries.

The study also revealed the main challenges encountered by the TITCK that act as barriers to a good-quality review system including limitations in human resources and physical and technological infrastructure. Questionnaire responses from the comparative agencies indicated that incomplete submissions and lack of experienced staff were considered barriers to an effective and efficient authority. The key features of the TITCK review process compared with TGA, Health Canada, SFDA, and HSA are summarized in Table 6 (Tables 2–6 have been combined as a Supplementary Material).

**Table 6:** Key features of the five agencies' review processes.

**Review feature**	**Turkey**	**Australia**	**Canada**	**Saudi Arabia**	**Singapore**
Certificate of Pharmaceutical Product is required at time of submission	✘	✘	✘	✓	✘
More than 20% of review staff are medically qualified	✓	✓	✘	✘	✘
The authority sets target time for scientific assessment	✘	✓	✓	✓	✓
The authority sets overall review and approval target time	✓	✓	✓	✓	✓
Questions to sponsors are batched at fixed points in the review	✘	✓	✘	✓	✓
Recording procedures allow company response time to be measured and differentiated in the overall processing time	✘	✓	✘	✓	✓
The authority recognizes medical urgency as a criterion for accelerating the review and approval process for qualifying products	✓	✘	✓	✓	✓
Quality, safety, and efficacy technical data sections are reviewed in parallel rather than sequentially	✘	✓	✓	✓	✓
Pricing discussions are separate from the technical review	✓	✓	✓	✘	✓
The focus is on checking quality in the market place and requirements for analytical work do not delay marketing authorization	✘	✓	✓	✓	✓

## Discussion

Enhancing patients' access to new medicines is of critical importance for all healthcare stakeholders, including regulatory authorities. However, this goal cannot be realized in many countries around the world due to barriers that include long approval timelines, increased payer pressures, and complicated legal practices. The comparison of various international regulatory systems and review processes can facilitate the identification of weaknesses and the sharing of best practices that may assist in overcoming some of these barriers.

As the second largest pharmaceutical market in Central/Eastern Europe (United States Department of Commerce, 2016), the Turkish regulatory health authority TITCK has sought over the past several decades to align its standards with those of other mature developed health agencies in order to ensure patients' timely access to medicines. The current study aimed to demonstrate the performance of the TITCK registration review model with those of other similarly sized developed regulatory agencies to identify areas of strength and those requiring further improvement in relation to the review process as well as to assess the level of adherence to good review practices in order to facilitate the TITCK progress toward this goal.

The comparative agencies of TGA, Health Canada, SFDA, and HSA were selected to ensure an adequate representation of health agencies with similar characteristics, review models and maturity in order to enlarge the geographic representation outside those of leading agencies in Japan, EU and US. The population size of Australia and Canada was also taken into consideration as was the position of the SFDA and HSA as leading agencies in the Middle East and Asia Pacific regions (Hashan et al., 2016).

### Review type and process

The TITCK currently performs a full review (type 3A) for all new active substances and a NAS marketing authorization application can be submitted in Turkey prior to any approval in the world although, a pre-approval by a reference regulatory agency is a prerequisite for final approval.

A complete GMP application and inspection process is a prerequisite for the application, but may be run in parallel for life-saving and critical products. Considering the limited resources within the TITCK and the relatively large number of applications received, the agency may wish to conserve constrained resources through the use of a risk stratification approach for the review (Alsager et al., 2015). With this approach, under certain circumstances, agencies such as TGA and HSA conduct abridged reviews of products that have been approved by one or two or more reference agencies respectively. This system facilitates the conservation of resources for a full review of products that have not been previously reviewed or of medicines associated with a high risk for patients.

Additionally, the TITCK could benefit from the use of joint reviews or the use of the assessment outcomes from other regulatory health authorities, especially for the review of the clinical portion of dossiers, thus reducing the review burden for TITCK assessors. This option could be explored with agencies of similar size and resources such as Health Canada, SFDA or TGA. Moreover, the TITCK could benefit equally from the use of the GMP assessment and accreditation processes of other regulatory health authorities. In May 2013, the TITCK applied for full membership in PIC/S, which was achieved in 2017 and this will facilitate this collaboration. PIC/S was established to harmonize global GMP accreditation procedures by setting common standards for the GMP process, providing related training to inspectors and developing required competencies for the assessment among regulatory authorities to increase mutual confidence [The Pharmaceutical Inspection Co-operation Scheme, (PIC/S), 2016]. In order to minimize delays in the authorization process, the TITCK may also wish to consider conducting the GMP process in parallel to the registration review procedure, which is similar to the procedures employed by Health Canada, HSA, SFDA and TGA.

### CPP or evidence of prior marketing authorization approval

In Turkey, the submission of a CPP or an evidence of approval elsewhere at the time of application is not required; however, a CPP or evidence of approval in EU, US or another country is required for final authorization. This is similar to countries such as Mexico and China, which also require proof of prior marketing authorization before final approval (McAuslane et al., 2009). Other agencies employ the use of alternate evidence of market authorization such as information from other agency websites. However, other mid-sized agencies in countries such as Australia, Canada, and Singapore do not require a CPP when performing a full assessment. Although the SFDA requires a legalized CPP for regulatory submissions, this is not mandated by the WHO.

It is suggested that the TITCK, which proceeds with the full review of dossiers submitted in parallel with other developed agencies like EMA and FDA, but relies on the approval of those agencies to grant its final approval, consider abolishing the need for a CPP or any evidence of prior marketing authorization approval. Marketing authorization application dossiers provided by global companies already meet the TITCK requirements that submitted data are in accordance with the ICH guidelines and the CTD format, including all five quality, non-clinical, and clinical modules since the content of the dossier with the exception of module one data is aligned with those of other developed agencies.

### Pricing

Like the SFDA, the TITCK requires information relating to pricing as part of the review process and this includes the reference price lists for the drug product in five countries, namely Portugal, Spain, France, Italy, and Greece. Comparatively, price evaluation is not part of the review process at TGA, Health Canada or Singapore. Although final marketing authorization approval does not depend on the pricing negotiation, other pre-marketing administrative steps such as final packaging and labeling approval and sales and importation permission to rely on prior price approval. Currently, price negotiation is a complex process in Turkey, where the price approval of a medicine does not include any scientific regulatory assessment but is subject to the evaluation and consensus agreement of stakeholders other than the TITCK within the FDK including the Social Security Institute, Ministry of Finance, and Under-Secretariat of Treasury as well as the Ministry of Development (Türkiye Ilaç ve Tibbi Cihaz Kurumu Price Decision, 2015). In the established agencies such as EMA, FDA, Health Canada and TGA, pricing is conducted as an independent separate process after marketing authorization approval and the agency is only responsible for the scientific regulatory assessment of the application and does not get involved with pricing or reimbursement discussions. Accordingly, it is suggested that the TITCK should not perform pricing assessment as part of the review process, but rather initiate the process separately and preferable in parallel or following licensing.

### Approval timing

From an industry perspective, the TITCK is generally perceived to have a relatively long approval timeline in comparison with other mature health authorities such as FDA and EMA, which consequently delay patients' access to medicines (Kanzik and Hincal, 2011). Study results indicated that the mean approval times for NAS marketing authorization at the TITCK were 350 working days (490 calendar days), 490 working days (476 calendar days) and 270 working days (378 calendar days) for the years 2013, 2014, and 2015 respectively despite an increase in the number of applications reviewed. These timings exceeded the agency's overall target time of 210 calendar days but this excludes companies' response time to questions, suggesting room for improved timeliness, consistency, and process predictability in the system.

However, the industry data (Figure 6) shows even longer review times, this does include company response time. Industry experience also shows that question and answer phases in dossier reviews can take 15 months, with an average of 10 to 15 questions received for each NAS application and an average of 2–4 months to close each question (based on TITCK official letters/Communication). It is suggested that the TITCK batch questions and set target response timelines for companies. Setting target timelines for question and answer phases and enhancing the dialogue and transparency between the TITCK and the industry could improve the quality of dossier submissions and reduce the number of agency questions raised during the review process.

Additionally, delays in approval may also be related to the structure and working procedures of the committees through which most review and assessment decisions are made. The TITCK may wish to consider delegating the review and assessment of some variations or extensions to internal assessors in order to reduce the number of dossiers assessed by committees and thus these groups could focus on new product applications and major clinical or quality variations.

The TITCK are planning to convert their manual tracking procedure for dossiers to an internal electronic system; however, currently, information regarding timelines and milestones are not available to stakeholders in a systematic formal way. Establishing an electronic tracking system with target timelines would enhance the efficiency and continuity of the review process while enabling the TITCK to monitor the timelines between milestones as well as to observe the time between first-in-world approval and approval in Turkey.

The TITCK has established target times for the authorization procedure and overall approval, whereas SFDA, TGA, Health Canada, and HSA set separate target times for validation, scientific assessment, and authorization as well as overall approval times. Defining target timing for individual milestones within the review facilitates planning for both agencies and sponsoring companies and therefore permits the identification of the most appropriate areas for improvement.

For 2012–2015, SFDA review times were shorter than target times, review times for TGA were longer than targets, and review times for Health Canada were approximately on target. Hashan and colleagues noted that Health Canada makes vigorous efforts to keep to target times in order to avoid penalties of up to 50% of user fees as mandated by the User Fees Act (Hashan et al., 2016). Industry data indicates that review and approval timing is longer in Turkey compared with Australia, Canada, Saudi Arabia, and Singapore, while for the TITCK the data did not include timing for question and answers and are also presented as means, thus making comparisons with the other agencies in this study difficult as they are presented as medians (Hashan et al., 2016).

### Good review practices

GRevP facilitate a timely and high-quality regulatory review and enhances global regulatory harmonization and convergence by facilitating the exchange of best practices, assessment reports and outcomes among regulatory authorities, which significantly contribute to the better management of regulatory resources and the timely approvals of medicines (World Health Organization, 2015). Thus previous studies demonstrated that building quality and GRevP into the regulatory review process is a significant regulatory performance indicator of the approval and timelines (Cone and McAuslane, 2006). In relation to this, the WHO has set the standards of GRevP with the aim to guide national and regional regulatory authorities (World Health Organization, 2015). Similar to other comparator agencies, the TITCK in this study employ many of the essential elements of GRevP. However, the GRevP implemented by the TITCK are not currently formalized and require an enhancement in some areas such as transparency to stakeholders, training tools such as induction courses for new assessors and building an electronic tracking system available to stakeholders. By adopting the standards of the global GRevP guidelines and monitoring their implementation within the TITCK, GRevP could be formalized to become a mandatory system to improve and ensure consistency, timeliness, and review process predictability.

The transparency of any regulatory health authority can be defined in terms of the ability and willingness of the agency to assign time and resources to provide information on its activities to both the informed public as well as healthcare professionals and the industry. Each regulatory authority may prioritize transparency differently depending on the main drivers and incentives to allocate time and resources for this goal including political will, public pressure and media attention on the regulatory review system. Additionally, regulatory agencies may aim to increase the level of confidence in their review systems in order to provide assurance regarding safety provisions and to ensure better staff morale and performance (McAuslane et al., 2009). The TITCK may wish to consider providing communication elements which could contribute to enhance the transparency of the review process as well as the quality of applications, such as the provision of a summary basis of approval; thus communicating the agency decision-making process to companies, patients, and healthcare providers. In providing a rationale for the publication of summary basis of decision document, Health Canada states on its website that this communication “… *improves the transparency of the drug and medical device regulatory review processes. They also give Canadians improved access to information about decisions to authorize products for sale in Canada*” (Health Canada, 2016).

Finally, the TITCK is not currently implementing a structured framework for the evaluation of the benefit-risk assessment of medicines, which is the key step in the review process. Thus, the assessment process depends largely on the reviewers' expertise and experience, which may vary significantly. Therefore, to enhance the quality and standardization of the review process, the TITCK has recently decided to implement a structured peer review process that is practiced by many mature agencies. For example, in Australia the TGA uses a multi-layered peer review process during which applications are reviewed for a second time by senior reviewers (Khalaf Al-Essa, 2011). The TITCK may wish to review the benefit-risk assessment templates of other regulatory authorities to gain insight into common practice, but because establishing a benefit-risk framework would require a high level of expertise and resource, it is suggested that the TITCK consider adopting and implementing the Universal Methodology for Benefit-Risk Assessment (UMBRA) framework, as this has been positively assessed by several mature agencies and is currently under evaluation by agencies in jurisdictions with emerging pharmaceutical markets (Walker et al., 2013).

The strengths of this approach to the evaluation of the TITCK is that the data were obtained directly from the most senior personnel within the agency who were very familiar with the advantages of the processes in place as well as its limitations. This was followed up by face-to-face interviews, which clarified the areas which needed certain additional information. The main advantage of the collected data is that they have allowed a comparison with other comparable agencies, which then enabled a series of recommendations to improve the review, which are provided. Several of these suggestions have now been initiated by the TITCK, while others are being discussed and prioritized. As an outcome of this work and the publication of these results, the agency has improved its transparency as well as communicating its strategy going forward to its stakeholders namely the Pharmaceutical Industry and patients.

### Recommendations

The comparison of the current TITCK processes and practices with those of similar medium-size regulatory agencies such as TGA, Health Canada, SFDA, and HSA has enabled the development of several proposals to assist the agency in its efforts to become an internationally recognized reference agency. It is suggested that the TITCK may wish to consider:

- Obviating delays caused by the current GMP processes by benefiting from the GMP inspection outcomes and GMP certificates issued by other authorities and by expediting the process of the mutual recognition as a member of the international organization of PIC/S to follow standard schemes in the GMP accreditation process.
- Expediting patients' access to medicines by removing the requirement for prior approval by a reference agency or a CPP.
- Conserving resources and reducing the time required for the review by exploring the possibility of introducing shared or joint reviews with other comparable regulatory authorities.
- Optimizing the review time and predictability, by batching the questions raised during the review process and set reasonable deadlines for companies to respond. This would enable the TITCK and companies to better plan their resources and maximize their efforts to reduce the clock stop period during the review of their applications.
- Reducing the overall approval times and the timing between first-in-world approvals and medicines' availability in Turkey by redefining the pricing process and separating it from marketing authorization.
- Facilitating a timely and high-quality regulatory review and enhancing global regulatory harmonization and convergence by the formal implementation and monitoring of GRevP.
- Improving their internal tracking and external stakeholder transparency and communication by defining target times for each review milestone in addition to the overall authorization timing.
- Improving transparency and communication by the development of publicly available summaries of the basis for approval.

## Author contributions

EM: designed the study, collated and analyzed the data, designed and presented the results and wrote the manuscript; SW: designed the study, collated and analyzed the data, designed and presented the results and wrote the manuscript; HG, AA, HC, and OK: facilitated the data collection and contributed to the manuscript.

### Conflict of interest statement

HG is the President, AA is the Vice President, HC is the Head of the Registration Department and OK is the President Advisor and Regulatory Coordinator of the Turkish Medicines and Medical Devices Agency (TITCK), SW is the founder of CIRS, London, UK and Professor of Pharmaceutical Medicine at Cardiff University and EM is a Doctoral Student at Cardiff University who conducted the research described in this manuscript.

## Abbreviations

CIS: Commonwealth of Independent States
CMC: chemistry, manufacturing, and controls
EMA: European Medicines Agency
FDK: Fiyat Degerlendirme Komitesi
GMP: good manufacturing processes
GRevP: good review processes
ICH: International Conference on Harmonization of Technical Requirements for the Registration of Pharmaceuticals for Human Use
MENA: Middle East and North Africa
PIC/S: Pharmaceutical Inspection Co-operation Scheme
TITCK: Türkiye Ilaç ve Tibbi Cihaz Kurumu
US FDA: United States Food and Drug Administration.
