# Supplementary material for: The Turkish Medicines and Medical Devices Agency: Comparison of Its Registration Process with Australia, Canada, Saudi Arabia, and Singapore
Source: Front Pharmacol. 2018 Jan 25;9:9. doi: 10.3389/fphar.2018.00009 (PMC5789679; doi:10.3389/fphar.2018.00009)
Supplement: Supplementary file 1 [file DataSheet1.pdf]

Characteristics of the five agencies

Quality measures

Measure	Regulatory authority				
	**Turkey (6/7)**	**Australia (6/7)**	**Canada (5/7)**	**Saudi Arabia (6/7)**	**Singapore (5/7)**
Internal quality policy	✓	✓	✗	✗	✗
Good review practice system	✓ *(Informally)*	✓ *(Informally)*	✓ *(Formally)*	✓ *(Informally)*	✓ *(Informally)*
Standard operating procedures for guidance of assessors	✓	✓	✓	✓	✓
Assessment templates	✓	✓	✓	✓	✓
Dedicated quality department	✓	✗	✗	✓	✗
Scientific committee	✓	✓	✓	✓	✓
Shared and joint reviews	✗	✓ *(Occasionally)*	✓ *(Occasionally)*	✓[a]	✓ *(Occasionally)*

Transparency and communication parameters

	**Turkey (4/9)**	**Australia (9/9)**	**Canada (8/9)**	**Saudi Arabia (5/9)**	**Singapore (6/9)**
Feedback to industry on submitted dossiers	✓	✓	✓	✗	✗
Details of technical staff to contact	✗ *(But some details available)*	✓	✓	✗	✓
Pre-submission scientific advice to industry	✗	✓	✓	✓	✓
Official guidelines to assist industry	✓	✓	✓	✓	✓
Industry can track progress of applications	✓ *(Based on ad hoc contact)*	✓	✓	✓	✓
Summary of grounds on which approval was granted	✗	✓	✓	✗	✗
Approval times	✗	✓	✓	✓	✓
Advisory committee meeting dates	✗	✓	✗	✗	✗
Approval of products	✓	✓	✓	✓	✓

Continuous improvement initiatives

	**Turkey (4/5)**	**Australia (4/5)**	**Canada (3/5)**	**Saudi Arabia (5/5)**	**Singapore (4/5)**
External quality audits	✗	✗	✗	✓	✗
Internal quality audits	✓	✓	✓	✓	✓
Internal tracking systems	✓	✓	✓	✓	✓
Reviews of assessors' feedback	✓	✓	✗	✓	✓
Reviews of stakeholders' feedback	✓	✓	✓	✓	✓

Training and education

	Turkey (7/8)	Australia (8/8)	Canada (8/8)	Saudi Arabia (7/8)	Singapore (8/8)
International workshops/ conferences	✓	✓	✓	✓	✓
External courses	✓	✓	✓	✓	✓
In-house courses	✓	✓	✓	✗	✓
On-the-job training	✓	✓	✓	✓	✓
External speakers invited to the authority	✓	✓	✓	✓	✓
Induction training	✗	✓	✓	✓	✓
Sponsorship of post-graduate degrees	✓	✓	✓	✓	✓
Placements and secondments in other regulatory authorities	✓	✓	✓	✓	✓

Key features of review processes

Review feature	Turkey	Australia	Canada	Saudi Arabia	Singapore
Certificate of Pharmaceutical Product is required at time of submission	✗	✗	✗	✓	✗
More than 20% of review staff are medically qualified	✓	✓	✗	✗	✗
The authority sets target time for scientific assessment	✗	✓	✓	✓	✓
The authority sets overall review and approval target time	✓	✓	✓	✓	✓
Questions to sponsors are batched at fixed points in the review	✗	✓	✗	✓	✓
Recording procedures allow company response time to be measured and differentiated in the overall processing time	✗	✓	✗	✓	✓
The authority recognizes medical urgency as a criterion for accelerating the review and approval process for qualifying products	✓	✗	✓	✓	✓
Quality, safety, and efficacy technical data sections are reviewed in parallel rather than sequentially	✗	✓	✓	✓	✓
Pricing discussions are separate from the technical review	✓	✓	✓	✗	✓
The focus is on checking quality in the market place and requirements for analytical work do not delay marketing authorization	✗	✓	✓	✓	✓

[a] *Shared and joint review with the Gulf Cooperation Council countries*
